# Left pulmonary artery banding to repair ipsilateral diffuse pulmonary arteriovenous fistula

**DOI:** 10.1186/1749-8090-7-77

**Published:** 2012-08-27

**Authors:** Takuya Hirata, Kentaro Akagi, Shiro Baba, Fumitoshi Tsurumi, Tomohiro Nakata, Tadashi Ikeda, Hiraku Doi

**Affiliations:** 1 Department of Pediatrics, Graduate School of Medicine, Kyoto University, 54 Kawahara-cho, Shogoin, Sakyo-ku, Kyoto City, Kyoto, 6068507, Japan; 2 Department of Cardiovascular Surgery, Graduate School of Medicine, Kyoto University, Kyoto, Japan

**Keywords:** Pulmonary arteriovenous fistula, Diffuse type, Pulmonary artery banding

## Abstract

Congenital pulmonary arteriovenous fistula (PAVF) is a rare disease which causes hypoxemia by shunting deoxygenated blood from the pulmonary artery into pulmonary venous return. Lung transplantation is the most effective therapy to treat severe, diffuse PAVF. However, the availability of lungs for transplantation is limited in most parts in the world. For patients with diffuse PAVF affecting only one side of the lungs, ipsilateral pulmonary artery banding (PAB) is an effective treatment, but not yet standard of care. We report successful treatment of a patient with diffuse left-sided PAVF with PAB. We believe that PAB is an effective therapy for severe unilateral PAVF and may serve as a bridge to lung transplantation.

## Background

Pulmonary arteriovenous fistulas (PAVF) are abnormal pulmonary fistulas between pulmonary arteries and veins (PAVFs), bypassing the capillary network requiring for gas exchange [1]. Deoxygenized blood is shunted through these fistulas into pulmonary venous return and causes hypoxemia. More than 80% of PAVF is congenital [2]. Other etiologies include chest trauma, infection and amyloidosis. In congenital PAVF, 70% of patients have a history of hereditary hemorrhagic telangiectasia (HHT) which is caused by mutations in Endoglin (HHT1) and ALK1 (HHT2) [2]. Clinically, PAVF is generally classified into two-types: focal and diffuse. The focal type is common and curable by various therapies such as embolization [3]. In contrast, the diffuse type is much rarer, more severe and therapy is limited. For patients with diffuse PAVF, it is not possible to occlude all PAVFs. Lung transplantation is, thus, inevitable for patients with severe, diffuse PAVF [4]. However, as the demand for lung transplant outpaces available grafts, options for treatment are limited to observation or supplemental oxygen therapy as these patients await transplant.

In 2010, left pulmonary branch artery banding was reported as an effective procedure for diffuse left-sided PAVFs and avoids the need for total resection of the left lung [5,6]. Despite the effectiveness of this procedure, unilateral pulmonary artery banding (PAB) is not commonly performed for ipsilateral diffuse PAVF. We report a patient with diffuse left-sided PAVFs with severe hypoxemia and hypotension who recovered dramatically following ipsilateral PAB.

## Case presentation

A nine-year old boy was transferred emergently to our hospital with the chief complaints of hemoptysis and convulsion in 2011.

**Figure 1:**
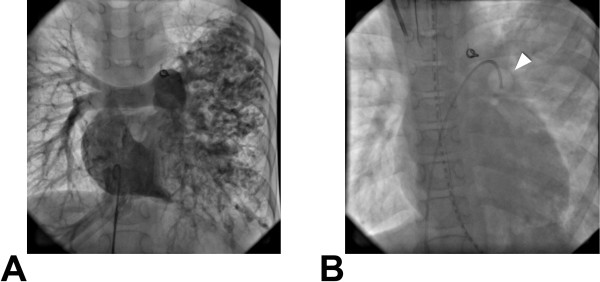
**Cardiac catheterization of our PAVF patient. A**) PAVFs localized in entire left lung by the right atrial angiography. The coil used for patent ductus arteriosus occulusion is shown in the center. **B**) An inflated balloon, indicated by a white arrowhead, localizes over the main trunk of left pulmonary artery.

Our patient had a history of repeated hospitalizations for pneumonia and initially presented with hypoxemia of 95% by pulse oximetry (SpO2) on room air. At the age of two years and three months, he was diagnosed as diffuse PAVF by computed tomographic angiography, pulmonary blood flow scintigraphy and cardiac catheterization. At that time, arterial blood gas showed partial pressure of arterial oxygen (PaO2) of 50 mmHg and arterial oxygen saturation (SaO2) of 82% on room air. PAVFs were localized only to the left lung. The number and size of the PAVFs increased over time (Figure 1A). Subsequently, the degree of right to left shunting increased and SpO2 decreased remarkably to about 75% on room air. To improve daily activity, home oxygen therapy was started at the age of six.

At nine years of age, the patient was then sent to our hospital emergently for hemoptysis and convulsions. The hemoptysis resolved spontaneously and the convulsions were controlled by intravenous midazolam. PaO2 was 50 mmHg and SaO2 was 68% at the time. Endotrachael intubation and mechanical ventilation was performed for airway protection and hypoxemia. Chest X-ray showed diffuse infiltration of the entire left lung. Magnetic resonance imaging showed a focal area of high signal intensity area in the occipital lobe, consistent with cerebral infarction and likely focus of the seizures. Despite maximal mechanical ventillatory support, PaO2 and SaO2 gradually decreased to less than 20 mmHg and 30%, respectively, despite 100% inspired fraction of oxygen (FiO2) on hospital day two. Systolic arterial blood pressure was decreased to less than 40 mmHg likely caused by myocardial ischemia. Given that the patient’s PAVFs were localized only in the left lung, we occluded the main trunk of left pulmonary artery (PA) with a Swan-Ganz catheter (Figure 1B). Immediately after inflating the balloon, PaO2 dramatically elevated from 22.6 mmHg to 44.9 mmHg and SaO2 elevated from 30% to 76%, respectively. Blood pressure also improved to 85 mmHg. When deflating the balloon again, PaO2 and SaO2 dropped quickly to 29.5 mmHg and 50% in 5 minutes, respectively. The Swan-Ganz catheter was left inflated to occlude the left PA. Left PAB was completed the following day on the left PA (circumference 15 mm).

**Figure 2:**
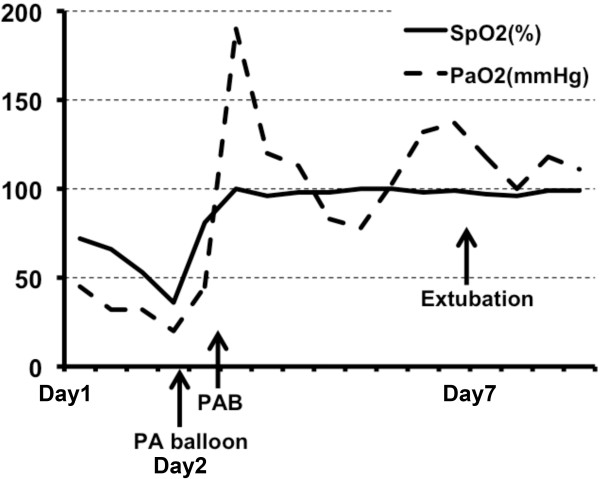
**Clinical course of the patient after our ICU admission.** SpO2 and PaO2 were dramatically elevated after PAB.

After left PAB, SpO2 and PaO2 remained approximately 100% and 100 mmHg, respectively, on 30% FiO2 (Figure 2). Blood pressure remained greater than 80 mmHg. The patient was successfully extubated and weaned to 3 L/min supplemental oxygen seven days after the left PAB.

Six months after the left PAB, SpO2 remains greater than 98% without supplemental oxygen. He is restricted to a wheel chair because of mild lower limb paralysis due to brain infarction, but he is able to attend school with moderate restrictions to physical activity.

## Conclusions

We report a successful case of unilateral PAB for a severe hypoxemia due to diffuse, ipsilateral PAVFs.

**Figure 3:**
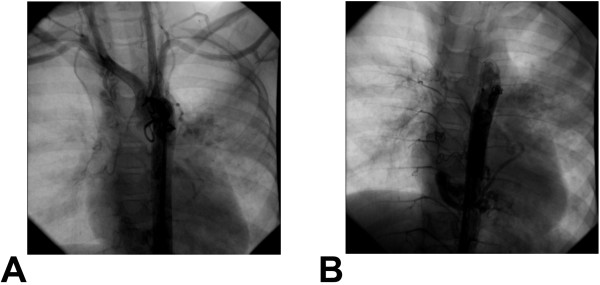
**Multiple collateral vasculature is seen from the pulmonary arteries directly to pulmonary veins and left atrium. A**) Ascending Aortography. **B**) Descending Aortography.

PAVF is especially rare in children and few cases of unilateral PAVF have been reported. Most cases require surgical intervention [1,2,4]. In cases of diffuse PAVFs, definitive surgical treatment is difficult or impossible given the large area of affected lung if not entirely affected. For these patients, unilateral pneumonectomy, unilateral lung transplantation or unilateral PAB is often inevitable. In our case, extensive collateralization due to persistent hypoxemia was observed angiographically (Figure 3). Unilateral PAB was recommended given inability to procure a donor lung and high risk of complications from pneumonectomy. The risk of bleeding complications during pneumonectomy was felt to be prohibitive given extensive collateralization of the vascular anatomy. In addition, the patient presented in cardiogenic shock in the setting of myocardial ischemia due to profound hypoxemia, absolutely precluding invasive surgery. The remarkable improvement in hemodynamics observed during transient left pulmonary artery occlusion with the Swan-Ganz catheter prompted us to consider and ultimately pursue complete left-sided PAB. The PAB was performed by careful and incremental ligation of the left pulmonary artery while monitoring PaO2. When PaO2 was normalized, we stopped tightening the PAB. Total occlusion of left pulmonary artery was avoided because of concerns for ischemic necrosis leading to systemic inflammation and further hemodynamic compromise. In addition, total PAB may make future lung transplantation more difficult or impossible. Minimally invasive PAB was thus the easiest and safest treatment option for our patient.

Two cases of PAB for PAVF have been reported, both of which were successful [5,6]. However, our patient underwent PAB with greater disease burden and was more critically ill at the time of the procedure than the reported patients. Unilateral PAB successfully rescued a severely decompensating patient with dramatic and durable recovery.

In conclusion, PAB is an effective, minimally invasive procedure for diffuse PAVF involving a large area of ipsilateral lung. We propose that this procedure is suitable for bridging use for lung trnsplantation. Long-term effectiveness and prognosis in this procedure is not known and requires further investigation.

## Consent

Written informed consent was obtained from this patient and her mother for anonymous publication of this case report and any accompanying images. A copy of the written consent is available for review by the Editor-in-Chief of this journal.

## Abbreviations

PAVF: Pulmonary arteriovenous fistula; PAVFs: Pulmonary fistulas between pulmonary arteries and veins; HHT: Hereditary hemorrhagic telangiectasia; PAB: Pulmonary artery banding; SpO2: Oxygen saturation of pulse oximetory; PaO2: Partial pressure of arterial oxygen; SaO2: Arterial Oxygen Saturation; FiO2: Inspired fraction of oxygen; PA: Pulmonary artery.

## Competing interests

The authors declare that they have no competing interests.

## Authors’ contributions

TH was a primary attending physician in the pediatric ward in Kyoto university hospital. KA was a secondary attending physician in the pediatric ward in Kyoto university hospital. SB was an attending physician in pediatric outpatient clinic in Kyoto university hospital, did left pulmonary artery occlusion test by a balloon catheter, and gave most of comments for this paper. FT was a technician in Kyoto university hospital catheter lab. TN was the second operator of the PAB. TI was an attending physician in the ward of cardiovascular department in Kyoto university hospital and the main operator of the PAB. HD was an attending physician in pediatric outpatient clinic and did left pulmonary artery occlusion test by a balloon catheter. All authors read and approved the final manuscript.

## Authors’ information

TH is a staff doctor and a pediatric cardiologist in Kyoto university hospital. KA is a pediatric cardiology residency in Kyoto university hospital. SB is an assistant professor and a pediatric cardiologist in Kyoto university hospital. FT is a catheter technician in Kyoto university hospital catheter lab. TN is an assistant professor and a pediatric cardiovascular surgeon in Kyoto university hospital. IT is an associate professor in Kyoto University and a pediatric cardiovascular surgeon in Kyoto university hospital. HD is an assistant professor and a pediatric cardiologist in Kyoto university hospital.
